# Nanoscale Secondary Ion Mass Spectrometry determination of the water content of staurolite

**DOI:** 10.1002/rcm.9331

**Published:** 2022-07-07

**Authors:** Samantha Azevedo‐Vannson, Laurent Remusat, Hélène Bureau, Keevin Béneut, Bernardo Cesare, Hicham Khodja, María Jiménez‐Mejías, Mathieu Roskosz

**Affiliations:** ^1^ Muséum National d'Histoire Naturelle, Institut de Minéralogie, de Physique des Matériaux, et de Cosmochimie (IMPMC), UMR CNRS 7590 Sorbonne Université Paris France; ^2^ Department of Geosciences University of Padova Italy; ^3^ LEEL, NIMBE, CEA, CNRS Université Paris‐Saclay France; ^4^ Institut des Sciences de la Terre d'Orléans (ISTO), UMR 7327 Université d'Orléans, CNRS, BRGM Orléans France; ^5^ Geosciences Barcelona (GEO3BCN‐CSIC), C/Lluis Solé i Sabarís s/n Barcelona Spain; ^6^ Instituto Geográfico Nacional, Centro Geofísico de Canarias Santa Cruz de Tenerife Spain

## Abstract

**Rationale:**

Staurolite is an important mineral that can reveal much about metamorphic processes. For instance, it dominates the Fe–Mg exchange reactions in amphibolite‐facies rocks between about 550 and 700°C, and can be also found at suprasolidus conditions. Staurolite contains a variable amount of OH in its structure, whose determination is a key petrological parameter. However, staurolite is often compositionally zoned, fine‐grained, and may contain abundant inclusions. This makes conventional water analysis (e.g., Fourier transform infrared (FTIR) spectroscopy or by chemical titration) unsuitable. With its high sensitivity at high spatial resolution, Nanoscale Secondary Ion Mass Spectrometry (NanoSIMS) is potentially a valuable tool for determining water contents in staurolite. However a calibration with relevant standards covering a large range of water content is required to obtain accurate and reliable analyses, because matrix effects typically prevent direct quantification of water content by SIMS techniques.

**Methods:**

In this study, a calibration for NanoSIMS analyses of water content by using minerals with crystallographic structures comparable to that of staurolite (i.e., amphibole and kyanite, an inosilicate and a nesosilicate, respectively) has been developed.

**Results:**

Water measurements in an inclusion‐free crystal from Pizzo Forno, Ticino, Switzerland, by FTIR spectroscopy (1.56 ± 0.14 wt% H_2_O) and by Elastic Recoil Detection Analysis (ERDA) (1.58 ± 0.15 wt% H_2_O) are consistent with NanoSIMS results (1.56 ± 0.04 wt% H_2_O).

**Conclusions:**

This implies that our approach can accurately account for NanoSIMS matrix effects in the case of staurolite. With this calibration, it is now possible to investigate variations in water content at the microscale in metamorphic minerals exhibiting high spatial variability and/or very small size (few micrometers).

## 1 INTRODUCTION

In metamorphic rocks water can be stored as the hydroxyl group (water that is structurally bound) in hydrous minerals like amphibole and talc in addition to nominally anhydrous minerals (NAMs) like pyroxene, garnet and rutile.^1^ One of these hydrated minerals is staurolite. Staurolite is a monoclinic nesosilicate with chemical formula (Fe,Mg,Zn,Co)_3–4_(Al,Fe)_17–18_(Si,Al)_8_O_48_H_3–4_.^2^ It is an index metamorphic mineral common in metapelites equilibrated in the lower amphibolite facies^3^ of Barrovian‐type metamorphism, where it is often associated with garnet and Al_2_SiO_5_ polymorphs.^4^ More rarely, it occurs in metapelites in the eclogite‐facies.^5^ Mg‐rich staurolite has been observed in high‐pressure metabasites,^6^ whereas Fe‐rich staurolite has been synthesized experimentally at suprasolidus conditions in metapelitic bulk compositions.^7^, ^8^ The crystal chemical formula of staurolite is not fully known to date, in particular as concerns its hydroxyl content.^2^, ^9^ Such variable OH content determines values between 1 and 2 wt% H_2_O in most reported staurolite analyses. Two types of reactions seem to control the water content of staurolite^10^: homogeneous reactions with cation‐hydroxyl substitutions and heterogeneous reactions with redox and dehydration equilibria. The latter appear to be favored by an increase in temperature.^9^ Staurolite has great significance during metamorphic processes. In common Ms‐Qz‐bearing metapelites it breaks down to garnet, biotite, and Al_2_SiO_5_, whereas the dehydration of staurolite in Qz‐absent protoliths may produce hercynitic spinel.^11^, ^12^ Little data exist concerning the water content of staurolite and its implications for metamorphic processes.^10^, ^11^ It is therefore necessary to collect more information on staurolite water contents in order to better understand its relevance in fluid control during metamorphic processes.

Nanoscale Secondary Ion Mass Spectrometry (NanoSIMS) is a powerful instrument for the determination of water contents at the micrometer scale in geological samples.^9^, ^13^, ^14^, ^15^, ^16^, ^17^, ^18^, ^19^ However, as with other SIMS instruments, several biases (e.g., instrumental fractionation), including the so‐called matrix effect,^20^, ^21^ need to be considered in order to obtain accurate quantitative results. During analysis, a primary ion beam of O^−^ or Cs^+^ sputters the surface of the sample. Secondary ions are emitted from the sample surface as a secondary ion beam, which is subsequently analyzed by a double focusing mass spectrometer. In order to calibrate measurements and obtain accurate results, the analysis of reference materials is required.^20^, ^21^ For a proper calibration, it is necessary to measure standards exhibiting the same or a similar matrix. This implies choosing standards sharing similar chemical composition^22^ and crystallographic structure with the samples, in order to obtain accurate data.

**FIGURE 1:**
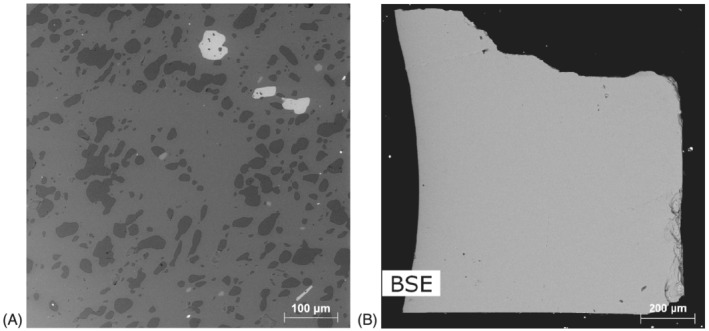
BSE images of A, staurolite from the Armorican massif (sample from the mineral collection of the Museum National d'Histoire Naturelle de Paris) and B, the staurolite from Pizzo Forno, Ticino, Switzerland used in this study. The Armorican crystal (A) contains many inclusions, unlike the mineral used in this study (B). FTIR and ERDA analyses of the Armorican crystal are hence more difficult

The set of standards must cover a wide range of water concentrations and must be minerals of gem quality, i.e., homogeneous and large enough to carry out multiple and independent analyses. However, there are few standards of staurolite that can satisfy these criteria, as they can be zoned or rich of inclusions^23^, ^24^ (Figure 1A). Hence, minerals with similar composition or crystallographic structure to those of staurolite can be used to define the calibration curve and thus to correct data for the matrix effect. This effect depends on the secondary ionization probability of a species (e.g., H) at the sample surface. In other words, it characterizes the emission yield of a given ion within different materials. We have chosen two minerals to test this approach: amphibole and kyanite. Both minerals, in particular kyanite, can be found in metamorphic rocks, and can be associated with staurolite at amphibolite‐facies conditions.^25^, ^26^ Amphibole, a monoclinic or orthorhombic inosilicate, can be encountered in plutonic and metamorphic rocks. Its chemical formula is: A B_2_ C_5_ T_8_ O_22_ W_2_ where A = Na, Ka, Ca, Li; B = Na, Li, Ca, Mn^2+^, Fe^2+^, Mg; C = Mg, Fe^2+^, Mn^2+^, Al, Fe^3+^, Mn^3+^, Ti^4+^, Li; T = Si, Al, Ti^4+^; W = (OH), F, Cl, O^2−^.^27^ Amphibole has a structure based on a double chain of tetrahedra and octahedra,^27^, ^28^ and has an O/OH ratio similar to that of staurolite. Kyanite, a triclinic nesosilicate, has a crystallographic structure very similar to that of the monoclinic staurolite.^25^, ^29^ In fact, the staurolite structure can be envisaged as an alternation between a kyanite module (Al_2_SiO_5_) and one of Fe_2_Al_0.7_O_2_(OH)_2_ composition along [010].^30^ In the second module, Fe^2+^ is in tetrahedral coordination. Hydrogen is linked to oxygens from octahedra to form OH groups. The strong structural analogy explains the frequent epitaxial intergrowths or replacements between the two minerals.^25^, ^31^

In this study, we present an original approach for correcting NanoSIMS measurements of water content in staurolite using kyanite and amphibole as standards. The accuracy of the corrected NanoSIMS measurements was evaluated by independent measurements of the same staurolite crystal by Fourier‐transform infrared (FTIR) spectroscopy and Elastic Recoil Detection Analysis (ERDA). The limitations of the NanoSIMS method and the influence of the crystal structures on the matrix effect are discussed.

## 2 MATERIALS AND METHODS

### 2.1 Samples

This study involves a crystal of staurolite from Pizzo Forno, Ticino, Switzerland,^32^ provided by the Museum of Mineralogy at the University of Padova, Italy (Figure 1B). It was prepared in three sections normal to each crystallographic axis. Samples were polished with diamond paste down to 0.25 μm. This staurolite crystal was analyzed by FTIR spectroscopy (on the plane (010)) and ERDA (on the plane (001)) (Table 1).

**TABLE 1:** FTIR parameters

	E//a	E//c
Thickness (μm) (*t*)	17.5 (± 0.5)	17.5 (± 0.5)
Integral absorbance (*A*)	299	173
Sum of absorbances	472	
*Ε*_i tot_ (l.mol.cm ^−2^)^a^	83,000 (±5000)	
Density (g/cm^3^) (*D*)^a^	3.76	

^a^ According to Koch‐Müller and Langer.^33^

**FIGURE 2:**
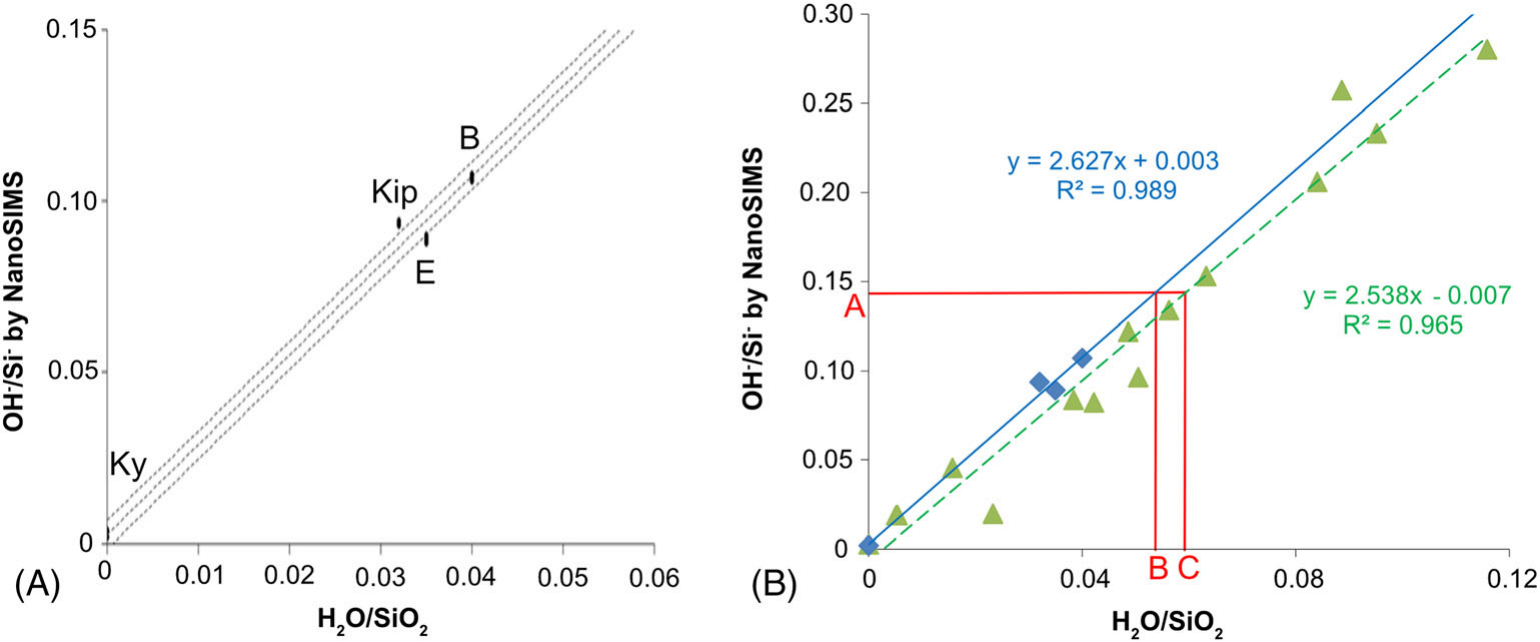
A, NanoSIMS calibration curve determined in this study representing the OH^−^/Si^−^ ratio measured by NanoSIMS versus H_2_O/SiO_2_. SiO_2_ contents are determined by the EPMA method and H_2_O contents by IRMS (see Deloule et al^34^ for amphiboles). Dashed lines represent the confidence interval and define the error of the measure. Only the middle line was used to calculate the water content of the staurolite. B = Bamble; Kip = Kipawa; E = Mont Emma; Ky = Kyanite. Bambe, Kipawa, and Mont Emma are amphiboles and contain 2.1 wt% H_2_O, 1.45 wt% H_2_O, 1.43 wt% H_2_O, and 0 wt% H_2_O, respectively. Ten measurements were made on the kyanite and on Bamble, six on Mount Emma, and five on Kipawa. B, Comparison of two calibrations: The blue curve represents the calibration defined using minerals (blue diamonds). The green dashed line represents the calibration determined by measurements of glasses (green triangles). For the OH^−^/Si^−^ ratio (= 0.145) measured by NanoSIMS on the staurolite crystal (A), the H_2_O/SiO_2_ obtained by (B) the mineral calibration is 0.054 versus 0.060 (C) with the glass calibration. Hence, water content obtained by mineral calibration is 1.56 (±0.04) wt% H_2_O and, by glass calibration, it is 1.72 (±0.05) wt% H_2_O. Water content is overestimated when using calibration based on glass measurements [Color figure can be viewed at wileyonlinelibrary.com]

One kyanite crystal and three amphiboles with known H_2_O/SiO_2_ ratios were used as standards to define the calibration curve (Figure 2A). These standards were polished with diamond paste without epoxy and embedded in pure indium. The H_2_O contents of these standards were measured by volumetry inside a vacuum line, where the volatiles are extracted by melting the sample. The extracted H_2_O is purified and measured. The SiO_2_ contents were measured by EPMA (Electron Probe MicroAnalysis).^34^ The H_2_O/SiO_2_ ratios of three amphiboles are between 0.032 and 0.04; kyanite is almost anhydrous (H_2_O <100 ppm) with a ratio H_2_O/SiO_2_ <0.0016.^35^ The kyanite crystal was provided by the mineral collection of the Muséum National d'Histoire Naturelle in Paris, France (ref. MIN2011‐3300). It was collected at la Pointe du Roucas Roux, at l'île du Levant, Var, France. Three different amphiboles (Mount Emma from Colorado, Kipawa from Quebec, and Bamble from Norway) were provided by Etienne Deloule from CRPG in Nancy, France.^34^ Kipawa and Bamble are magnesio‐hastingsites and Mont Emma is a pargasite^34^ and all are monoclinic.

### 2.2 NanoSIMS measurements

One polished section (001) of the staurolite crystal without resin was stuck on a double‐sided copper tape and analyzed with the Cameca NanoSIMS 50 installed at the Muséum National d'Histoire Naturelle in Paris. Three other polished sections of the staurolite crystal were analyzed to characterize the impact of the crystal orientation on the measurements. Each sample was polished to a quarter micrometer with alcohol and cleaned with ethanol in an ultrasonic cleaner. All samples and standards were gold coated (20 nm thick) before NanoSIMS analysis. Their surfaces were rastered by a 16 keV Cs^+^ primary beam, set to 23 pA (probe size around 200 nm). Secondary ions were recorded in multicollection mode: ^12^C^−^, ^16^OH^−^, and ^28^Si^−^, using electron multipliers with a 44 ns dead time. The mass resolving power was set to 8000, sufficient to resolve any interferences on the recorded masses. A flooding electron gun with a current of 8009 V was used for charge compensation. Measurement of ^12^C^−^ attested that analyses were not made at the edge of the sample or on a crack or a hole at the sample surface. Presputtering was carried out over a surface area of 5 × 5 μm^2^ for 300 s with a 200 pA primary Cs^+^ beam to remove surface contamination, gold coating, and to reach a steady‐state sputtering regime.^36^ Analyses were made on a 3 × 3 μm^2^ surface area during 100 cycles of 1.24 s each for a total measurement time per point of 431 s. Counts were collected only from the inner 1 × 1 μm^2^ using the beam blanking mode to reduce contamination from the edge of the area of interest.^17^ During the session, the vacuum never exceeded 3 × 10^−10^ Torr in the analysis chamber.

### 2.3 FTIR spectroscopy measurements

Fourier‐transform infrared (FTIR) spectroscopy is a non‐destructive method, which has a low detection limit^37^ (<1 ppm H_2_O). It is straightforward to identify traces of epoxy, for example, and to determine the speciation of water. The main drawback of this technique is the demanding sample preparation needed to obtain a doubly polished thin section for analysis in transmission mode. Polishing defects may affect the IR signal and the thickness of the doubly polished thin section determination uncertainties, and thus the error of the result. To perform analyses and evaluate the total integrated absorbance, the mineral was prepared along each crystallographic axis. For the staurolite there is absorbance only in E//a and E//c.^33^ Hence, only the (010) plane was studied. All analyses were carried out at the spectroscopy platform of the Institut de Minéralogie, de Physique des Matériaux et de Cosmochimie (IMPMC) at Sorbonne Université, France, using a Bruker IFS66v/s IR spectrometer under vacuum working with a homemade chamber composed of two Cassegrain Objectives. Measurements were made on a 17.5 (±0.5) μm thick doubly polished thin section with a spot size of 120 μm at the focal point. The section thickness was measured by scanning electron microscopy (SEM). Spectra were obtained between 600 and 7000 cm^−1^ in transmission mode with an aperture size of 300 μm. Typically, 256 scans were collected for each spectrum with a spectral resolution of 4 cm^−1^ and all analyses were made by polarized IR light perpendicular to the section (010). Each spectrum was normalized to the thickness of the section and corrected for the baseline, which was defined as a straight line between 3800 and 3200 cm^−1^. For IR measurements, the Beer–Lambert law (*A*_*i*_ *= ε*_*i*_ *·t·c*, where *A*_*i*_ is integrated absorbance, *ε*_*i*_ is the integrated molar absorption coefficient, *t* is the thickness, and *c* is concentration) is commonly used to define water concentration. In this study, the calibration determined by Koch‐Müller and Langer^33^ was applied with the equation: *c*_H2O_ (wt%) = (1.8·*A*_i.tot_)/(*D·ε*_i.tot_.*t*), where *D* is density (g/cm^3^), *A*_i.tot_ is measured or corrected total integrated area under the spectrum; *ε*_i.tot_ is the average total integrated absorption coefficient, which is equal to 83,000 ± 5,000 L·mol_H2O_^−1^ cm^−2^ according to Koch‐Müller and Langer;^33^ and *t* is the section thickness (cm). The total relative uncertainty is 10%. All parameters are summarized in Table 1.

### 2.4 ERDA measurements

Elastic Recoil Detection Analysis (ERDA) is a non‐destructive and reliable method to quantify H contents in minerals and glasses.^8^, ^38^, ^39^, ^40^ Analyses were made at the Laboratoire d'Etude des Elements Légers, CEA, Saclay, France, following well‐established procedures.^39^ A 2.8 MeV ^4^He^+^ incident beam with a 500 pA current is produced by a 3.75 MV Van de Graaf single‐stage accelerator and focused on a 3 × 3 μm^2^ surface. Three detectors are used simultaneously: an X‐ray detector to record particle‐induced X‐rays, an annular detector to record Rutherford back‐scattered particles, and an ERDA detector to record protons ejected from the sample through elastic collisions. An 11 μm Al foil transparent to energetic protons is mounted in front of the ERDA detector to stop scattered ^4^He^+^. The sample holder may be rotated either perpendicular to the beam in the standard Rutherford‐backscatter analysis geometry, or at a grazing angle of 15° from the incident beam for the ERDA configuration, resulting in a 12 × 3 μm^2^ incident beam. The beam is mapped on large areas (200 × 200 μm^2^ typically) of the sample surface during a single analysis for 3600 s. Multi‐elemental maps obtained simultaneously from Particle‐Induced X‐Ray emission (PIXE), Rutherford backscattering spectrometry (RBS) and ERDA are processed in order to locate and exclude any heterogeneities (i.e., grain boundaries, inclusions) or defects that would possibly result in an error in H content. The analytical procedure is described in detail elsewhere.^39^

## 3 RESULTS

**FIGURE 3:**
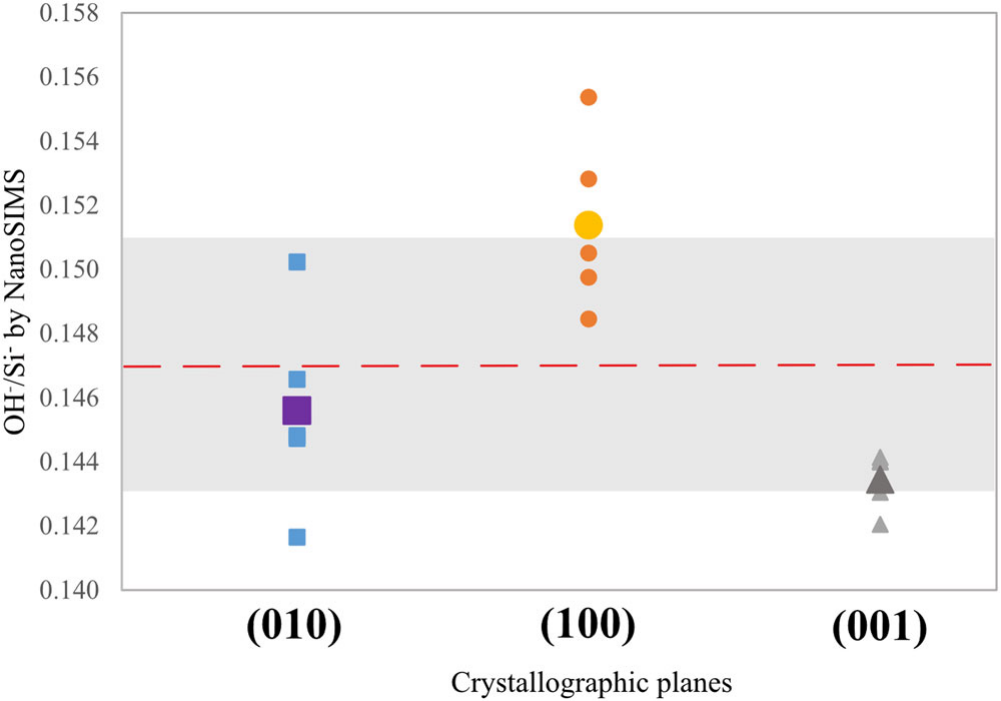
Measurement of the OH/Si ratio by NanoSIMS along the sections (010), (100), and (001) of the staurolite. On each section five analyses were made (analytical error for each single measurement is smaller than the size of the symbol). The largest symbols, square, circle, and triangle, correspond respectively to the mean values of each analysis group. The dashed line is the mean value of all analysis and is equal to 1.47 ± 0.04 × 10^−1^. The grey area represents the error on the mean value [Color figure can be viewed at wileyonlinelibrary.com]

The (Nano)SIMS measurements may be affected by crystallographic orientation. Deloule et al^34^ showed, for instance, the effect of the orientation of muscovite on D/H instrumental mass fractionation (e.g., poor reproducibility). To assess the potential influence of staurolite orientation on NanoSIMS analyses, the OH^−^/Si^−^ ratios were measured along the three crystallographic orientations (//a, //b and//c) of the same staurolite crystal. Five measurements spaced 15 μm apart were carried out on each section. The measurements of the OH^−^/Si^−^ ratios yield similar values of 0.147 ± 0.004. The error corresponds to the standard deviation defined over the 15 measurements. The dispersion is only 2.8% for all measurements (Figure 3). We thus conclude that, in the case of staurolite, NanoSIMS is insensitive to orientation effects for water concentration measurements. Hence, it will provide accurate results on any crystallographic orientation of the sample.

Data collected on the three amphiboles and the kyanite standards determine a consistent calibration curve (Figure 2A). The H_2_O concentration calculated from NanoSIMS measurements of OH^−^/Si^−^ ratios is 1.56 (± 0.04) wt% (Table 2). The uncertainty quoted here is derived from the standard error of the mean.

**TABLE 2:** Results of staurolite water content according to different methods

Methods	Water concentrations [H_2_O] (wt%)	±^b^
FTIR^a^	1.56	0.14
NanoSIMS	1.56	0.04
ERDA	1.58	0.15

^a^ *c*_H2O_ (wt%) = (1.8*A)/(D*E*t); according to Koch‐Müller and Langer.^33^

^b^ See the text for further information about the error calculations.

The ERDA map recorded over our staurolite sample shows a homogenous distribution of hydrogen in the crystal at the scale of a few micrometers. The H_2_O concentration determined by ERDA is 1.58 (±0.15) wt% (Figure 2; Figure S1, supporting information; and Table 2).

**FIGURE 4:**
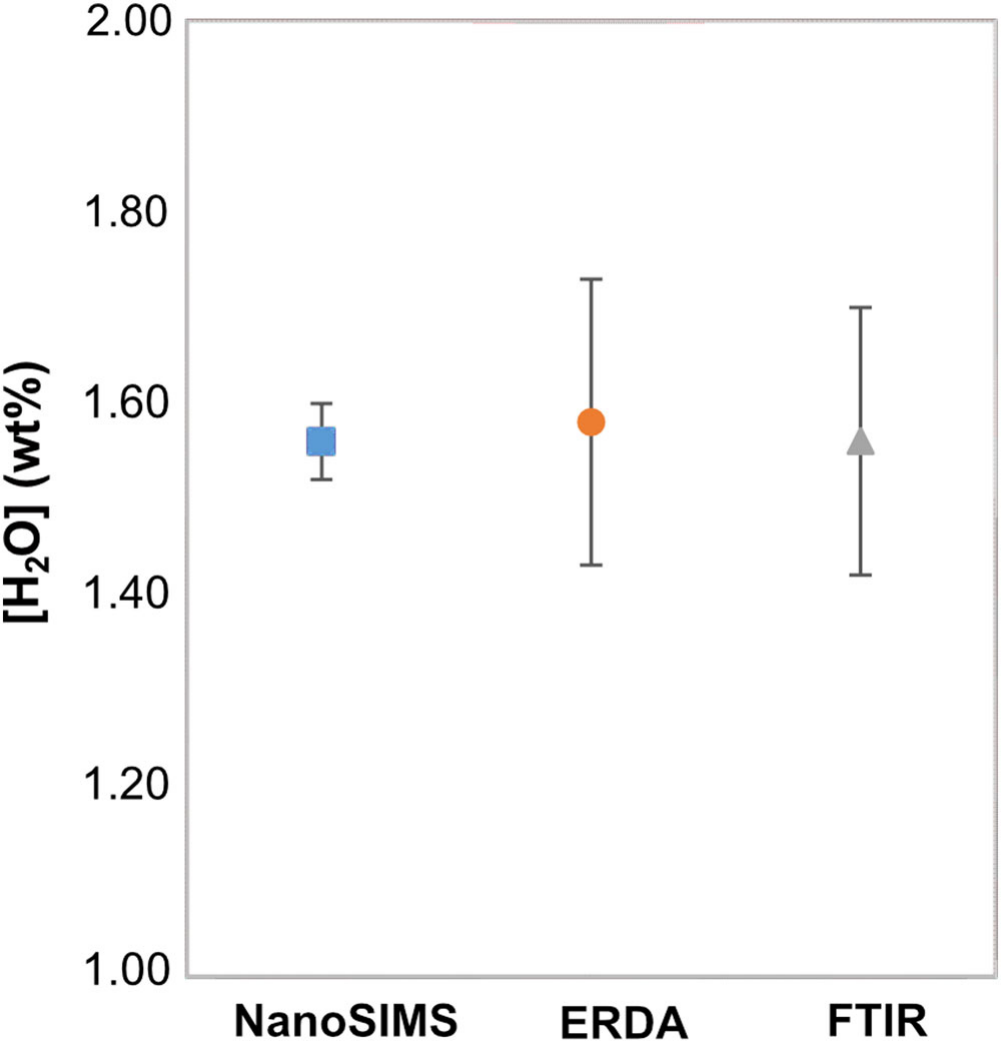
Comparison of water concentrations in staurolite obtained by the three methods FTIR, ERDA and NanoSIMS. Results are similar with 1.56 (± 0.14), 1.58 (± 0.15), and 1.56 (± 0.04) wt% H_2_O, respectively [Color figure can be viewed at wileyonlinelibrary.com]

Polarized spectra of the staurolite section (010) determined by the FTIR method are similar to those previously reported in the literature.^8^, ^33^, ^38^, ^39^, ^40^, ^41^ They show typical bands at 3345 cm^−1^, 3460 cm^−1^, 3580 cm^−1^, and 3680 cm^−1^ (Figure S2, supporting information), which correspond to three crystallographically different OH groups with diverse proton positions (H1; H2; H3).^41^, ^42^ Each spectrum depends on crystallographic direction. To determine water contents in staurolite by FTIR spectroscopy, the analysis has to be made on the (010) section (perpendicular to the b‐axis). Along this plane, the two perpendicular crystallographic axes a and c were investigated. The water concentration was derived from the total absorbance and the normalization was made from the integrated absorbance. The H_2_O concentration recalculated from FTIR spectroscopy was found to be 1.56 (± 0.14) wt% H_2_O (Figure 4; Table 2).

Hence, the determination of water content by NanoSIMS appears consistent with FTIR spectroscopy and ERDA. Furthermore, NanoSIMS has a better precision and measurements are made on a smaller sample volume.

## 4 DISCUSSION

We have shown here that NanoSIMS, ERDA and FTIR spectroscopy provide consistent determinations of the water content of a staurolite crystal with an average value of 1.57 (±0.15) wt% H_2_O (Figure 4; Table 2). All analyses give similar results showing the reliability of our approach for correcting NanoSIMS data on staurolite using kyanite and amphibole as standards. NanoSIMS has many advantages (e.g., analyses at micrometer scale, precision) that make it relevant and complementary to FTIR or ERDA. Two aspects of this method are further discussed here as well as the influence of crystal structure on matrix effect.

### 4.1 Ion emissivity and matrix effect

When samples show variable SiO_2_ wt%, it is necessary to present the OH^−^/Si^−^ ratio as a function of the H_2_O/SiO_2_ ratio to obtain reliable values (Figure 2A). For instance, Bellatreccia et al^43^ used minerals as standards and not glasses, and applied this method (i.e., OH^−^/Si^−^ vs H_2_O/SiO_2_) to define a calibration curve. Like Thomen et al,^36^ we can define a simple model of secondary ion currents where:

(1) $$OH^{-}=I_{p}\cdot Y\cdot \left[\mathrm{OH}\right]\cdot \alpha_{\mathrm{OH}}^{-}\cdot T_{\mathrm{OH}}^{-}$$

(2) $$Si^{-}=I_{p}\cdot Y\cdot \left[\mathrm{Si}\right]\cdot \alpha_{\mathrm{Si}}^{-}\cdot T_{\mathrm{Si}}^{-}$$

where *I*_*p*_ corresponds to the current density of the primary beam, *Y* is the total sputtering yield, [OH] and [Si] are the surface densities of corresponding atoms. *α* represents the ionization probability of OH^−^ and Si^−^. Finally, *T*_OH_^*−*^ and *T*_Si_^*−*^ are transmission factors for OH^−^ and Si^−^, respectively. With Equations (1) and (2), the OH^−^/Si^−^ ratio can be expressed as:

(3) $$\frac{OH^{-}}{Si^{-}}=\frac{\left[\mathrm{OH}\right]}{\left[\mathrm{Si}\right]}\cdot \frac{\alpha_{\mathrm{OH}}^{-}}{\alpha_{\mathrm{Si}}^{-}}\cdot \frac{T_{\mathrm{OH}}^{-}}{T_{\mathrm{Si}}^{-}}$$

*T* depends on the NanoSIMS optics and the setting for the analytical session, while *α* is characteristic of the sample. The $\frac{\alpha_{\mathrm{OH}}^{-}}{\alpha_{\mathrm{Si}}^{-}}$ ratio defines the matrix effect and does not vary due to the similarity between the standards used to form the calibration curve. And, since instrument settings (e.g., slit size and position, detector settings …) do not change in a single session, the $\frac{T_{\mathrm{OH}}^{-}}{T_{\mathrm{Si}}^{-}}$ ratio remains constant during the analysis. Then, we obtain:

(4) $$\frac{OH^{-}}{Si^{-}}=\beta \frac{\left[\mathrm{OH}\right]}{\left[\mathrm{Si}\right]}$$

where *β* corresponds to the slope value of the calibration curve and depends only on the instrumental parameters and the emissivity of ions, which in turn are constant from one sample to another during the same session. If all standards are aligned on the calibration curve, then the matrix effect can be considered as corrected. Hence, with a simple model of secondary ion current, it is possible to show the link between the measured ion ratios and the true elemental ratios (i.e., $\frac{OH^{-}}{Si^{-}}=\beta \frac{\left[\mathrm{OH}\right]}{\left[\mathrm{Si}\right]}$ and not $\frac{OH^{-}}{Si^{-}}=\beta^{\prime }\left[\mathrm{OH}\right]$). Indeed, *β’* depends, like *β*, not only on the instrumental parameters and the emissivity of ions, but also on the SiO_2_ content, which can vary between two samples. This is crucial here, as staurolite has a lower SiO_2_ content (28.77 wt%) than our standards (amphibole with SiO_2_ content between 40.87 and 50.98 wt% and kyanite 36%). The calibration used here (OH^−^/Si^−^ as a function of H_2_O/SiO_2_) is the most suitable for analyzing a set of samples with variable SiO_2_ contents.

### 4.2 Influence of standard composition and structure on matrix effect

The crystal structure affects SIMS measurements. Calibrations are thus ideally carried out on a set of materials with similar structure as the target samples and spreading over a significant range of concentrations of water content. However, this is not always possible for small, or exotic, mineral phases for which no good standards can be synthesized in the laboratory or found in nature. In the present study, we have investigated the possibility of using amphiboles and kyanite, exhibiting similarities in their structure, to calibrate for water content in staurolite measurements by NanoSIMS.

The similarity between staurolite and amphibole (for O/OH contents) or kyanite (for structure) is likely responsible for their comparable matrix effect with respect to SIMS measurements of OH^−^ with the Cs source of the NanoSIMS instrument. The matrix effect is expected to depend not only on the concentration of the analyzed element, but also on the concentration of the surrounding elements.^44^ Hence, the chemical composition and the structural organization of atoms are considered to control the matrix effect.^20^, ^45^ The matrix effect is expected to be small for elements like F, S, and Cl, whereas, for light elements like H, it should be more pronounced. Furthermore, concerning hydrogen, the matrix effect is more significant for samples with large hydrogen contents.^20^, ^46^ In kyanite, there is no hydrogen, whereas, for amphibole and staurolite, hydrogen is bonded to the oxygen of either the tetrahedron or the octahedron, respectively, to constitute OH groups. Hence, each OH group is surrounded by tetrahedra and octahedra mainly containing Si and Al. Thus, either the chemical composition of these minerals, or their similar crystal structures, determine the similar matrix effects observed in NanoSIMS determination of hydrogen.

### 4.3 Corrections using mineral standards vs corrections using glass standards

Crystal structure affects SIMS measurements. Calibrations are thus ideally carried out on a set of materials with similar structures to the target samples. The matrix effect is expected to depend not only on the concentration of the analyzed element, but also on the concentration of the surrounding elements.^44^ Hence, the chemical composition and the structural organization of atoms are considered to define the matrix effect.^20^, ^45^ Although the crystal structures of kyanite, amphibole, and staurolite are not identical, there are enough similarities (e.g., each OH group is surrounded by tetrahedra and octahedra mainly composed by Si and Al) between these minerals to accurately correct data for the matrix effect.

On the other hand, glasses are often considered as suitable standards for mass spectrometry in geochemistry and are often considered as versatile materials for SIMS calibration.^8^, ^47^, ^48^ We report in Figure 2B the comparison of results obtained by correction defined by minerals on the one hand and glasses on the other. The glasses used here are: STR9/STR10/STR11/STR13 shoshonite lavas from the Stromboli volcano^39^, ^49^ and a set of synthetic basaltic glasses with SiO_2_ between 44 and 50 wt%,^50^ containing a wide range of H_2_O between 0.03 and 5.7 wt%. Note that the silica content of these glasses is comparable to those of our crystalline compounds. Using glasses as standards, the staurolite H_2_O content is overestimated (1.72 ± 0.05 wt%). As in analyses of oxygen isotopes,^51^ this emphasizes the influence of a well‐crystallized material on the matrix effect compared to an amorphous material such as a glass, even in case of similar chemical composition. However, the chemistry is important, even if its effect seems minor in this study.

## 5 CONCLUSIONS

This study reports an original calibration method for NanoSIMS measurements of H in silicate minerals and its implications for determining water contents of staurolite. (1) During analysis, the sample orientation has a negligible impact on the OH^−^/Si^−^ ratio and, thus, on the hydrogen determinations. (2) Corrections established with the calibration based on amphibole and kyanite crystals result in an H_2_O content consistent with independent estimates by FTIR spectroscopy and ERDA. Although the crystallographic structures of inosilicate and nesosilicate are not identical, there are enough similarities between these minerals to correct for matrix effects in staurolite and hence to provide accurate and precise results. This approach strengthens the capability of NanoSIMS to investigate water concentration gradients at the micrometer scale and the determination of hydrogen contents in small crystals of staurolite.

### PEER REVIEW

The peer review history for this article is available at https://publons.com/publon/10.1002/rcm.9331.

## Supporting information


**Figure S1:** ERDA spectrum of the staurolite, Number of H counts versus Channels (i.e. Energy from 0 to 900 KeV). The area at the centre of the spectrum, from channel 100 to channel 240, corresponds to low energy H atoms, which originated from the bulk sample, and is used for the calculation of the H concentration. The area of the high energy peak corresponds to surface contamination (i.e. high energy H atoms). B. 200*200 μm^2^ map of the Deep H area, which is chemically quite homogeneous. Please note that the pixel size corresponds to 3 μm long by 12 μm wide.
**Figure S2:** Polarized spectra of the staurolite plane (010). Main bands are at 3345 cm^−1^, 3460 cm^−1^, 3580 cm^−1^ and 3680 cm^−1^ for E //c. Main bands are at 3460 cm^−1^, 3580 cm^−1^ and 3680 cm^−1^ for E//a. The OH absorption band of the E//a spectrum shows a slight oversaturation, that is included in the error bar.

## Data Availability

The data that support the findings of this study are openly available in “NanoSIMS determination of the water content of staurolite” at https://doi.org/10.48579/PRO/SNGYCN.
