# Supplementary material for: Nanoscale Secondary Ion Mass Spectrometry determination of the water content of staurolite
Source: Rapid Commun Mass Spectrom. 2022 Jul 7;36(17):e9331. doi: 10.1002/rcm.9331 (PMC9540019; doi:10.1002/rcm.9331)
Supplement: Supplementary file 1 — Figure S1: ERDA spectrum of the staurolite, Number of H counts versus Channels (i.e. Energy from 0 to 900 KeV). The area at the centre of the spectrum, from channel 100 to channel 240, corresponds to low energy H atoms, which originated from the bulk sample, and is used for the calculation of the H concentration. The area of the high energy peak corresponds to surface contamination (i.e. high energy H atoms). B. 200*200 μm2 map of the Deep H area, which is chemically quite homogeneous. Please note that the pixel size corresponds to 3 μm long by 12 μm wide. Figure S2: Polarized spectra of the staurolite plane (010). Main bands are at 3345 cm−1, 3460 cm−1, 3580 cm−1 and 3680 cm−1 for E //c. Main bands are at 3460 cm−1, 3580 cm−1 and 3680 cm−1 for E//a. The OH absorption band of the E//a spectrum shows a slight oversaturation, that is included in the error bar. [file RCM-36-e9331-s001.docx]

**Supplementary 1**


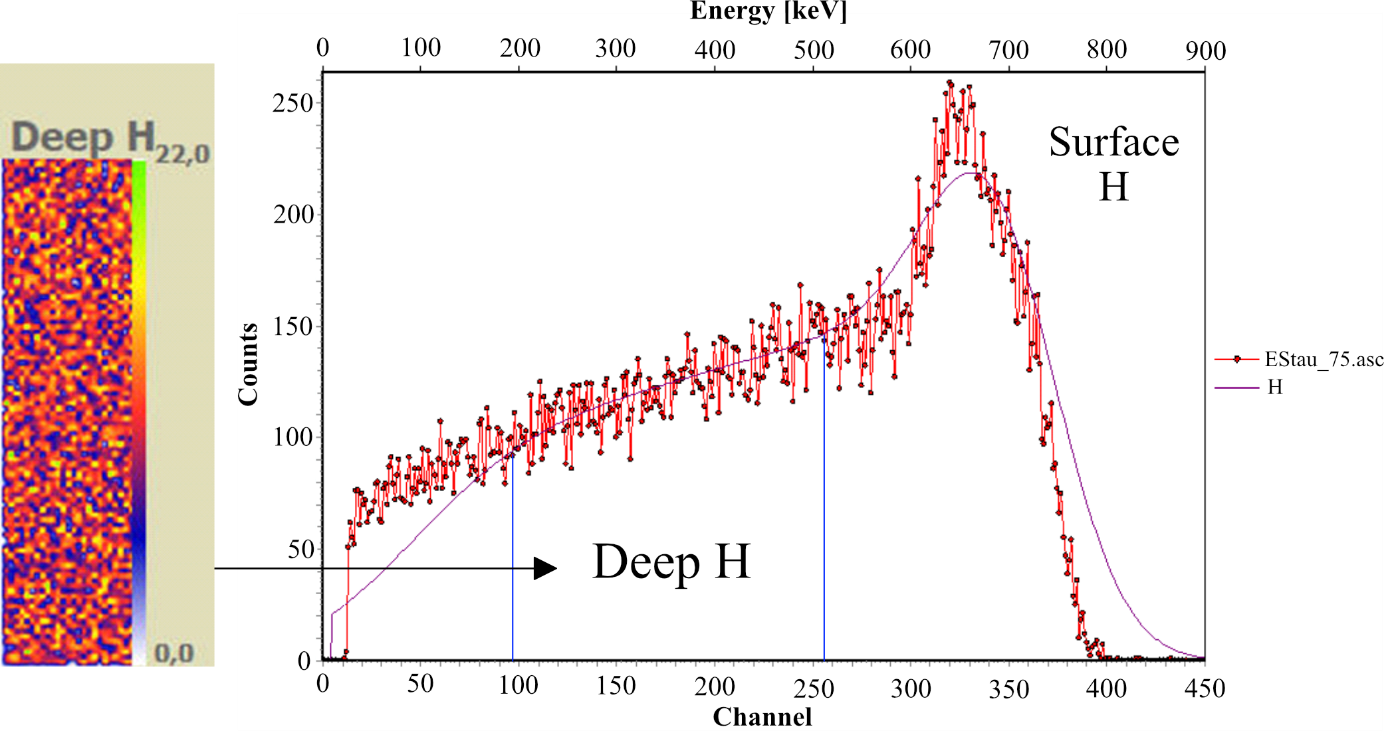


Figure S1: ERDA spectrum of the staurolite, Number of H counts versus Channels (i.e. Energy from 0 to 900 KeV). The area at the centre of the spectrum, from channel 100 to channel 240, corresponds to low energy H atoms, which originated from the bulk sample, and is used for the calculation of the H concentration. The area of the high energy peak corresponds to surface contamination (i.e. high energy H atoms). B. 200*200 µm2 map of the Deep H area, which is chemically quite homogeneous. Please note that the pixel size corresponds to 3µm long by 12 µm wide.


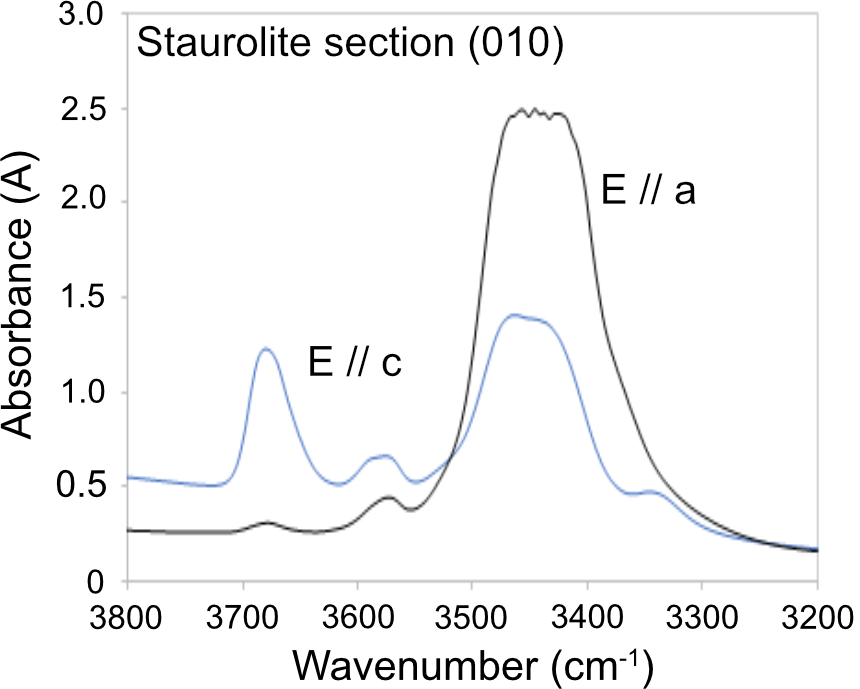


Figure S2: Polarized spectra of the staurolite plane (010). Main bands are at 3345 cm-1, 3460 cm-1, 3580 cm-1 and 3680 cm-1 for E // c. Main bands are at 3460 cm-1, 3580 cm-1 and 3680 cm-1 for E // a. The OH absorption band of the E//a spectrum shows a slight oversaturation, that is included in the error bar.
